# Accuracy assessment of target tracking using two 5-degrees-of-freedom wireless transponders

**DOI:** 10.1007/s11548-019-02088-9

**Published:** 2019-11-14

**Authors:** Roeland Eppenga, Koert Kuhlmann, Theo Ruers, Jasper Nijkamp

**Affiliations:** 1 Department of Surgical Oncology, The Netherlands Cancer Institute, Plesmanlaan 121, 1066 CX Amsterdam, The Netherlands; 2 Nanobiophysics Group, Faculty TNW, University of Twente, Enschede, The Netherlands

**Keywords:** Surgical navigation, Electromagnetic tracking, Wireless tracking, Surgical oncology, Tumor motion

## Abstract

**Purpose:**

Surgical navigation systems are generally only applied for targets in rigid areas. For non-rigid areas, real-time tumor tracking can be included to compensate for anatomical changes. The only clinically cleared system using a wireless electromagnetic tracking technique is the Calypso^®^ System (Varian Medical Systems Inc., USA), designed for radiotherapy. It is limited to tracking maximally three wireless 5-degrees-of-freedom (DOF) transponders, all used for tumor tracking. For surgical navigation, a surgical tool has to be tracked as well. In this study, we evaluated whether accurate 6DOF tumor tracking is possible using only two 5DOF transponders, leaving one transponder to track a tool.

**Methods:**

Two methods were defined to derive 6DOF information out of two 5DOF transponders. The first method uses the vector information of both transponders (TTV), and the second method combines the vector information of one transponder with the distance vector between the transponders (OTV). The accuracy of tracking a rotating object was assessed for each method mimicking clinically relevant and worst-case configurations. Accuracy was compared to using all three transponders to derive 6DOF (Default method). An optical tracking system was used as a reference for accuracy.

**Results:**

The TTV method performed best and was as accurate as the Default method for almost all transponder configurations (median errors < 0.5°, 95% confidence interval < 3°). Only when the angle between the transponders was less than 2°, the TTV method was inaccurate and the OTV method may be preferred. The accuracy of both methods was independent of the angle of rotation, and only the OTV method was sensitive to the plane of rotation.

**Conclusion:**

These results indicate that accurate 6DOF tumor tracking is possible using only two 5DOF transponders. This encourages further development of a wireless EM surgical navigation approach using a readily available clinical system.

## Introduction

In surgical oncology, the primary goal is to completely remove the tumor while sparing as much surrounding healthy tissue as possible. Tumor borders can be defined before surgery on diagnostic images acquired with modalities such as magnetic resonance imaging (MR) or computational tomography (CT). However, during the surgical procedure, surgeons generally have to translate this preoperative anatomical information to the actual anatomy.

Surgical navigation systems can be used to assist the surgeon in making that translation, improving the accuracy of tumor localization and tumor border assessment [1–4]. These systems register preoperative images to the actual surgical field, and by real-time projection of tracked surgical tools onto these images, the surgeons can navigate the targets. Many navigation systems rely on the assumption that the preoperatively acquired images apply to the real-time anatomy during surgery, i.e., they assume rigid anatomy [5–8]. However, in non-rigid target areas such as the breast and the abdominal area, there are vast intraoperative deformations caused by breathing, organ deformation and surgical manipulation [2]. These deformations can cause large tumor motions, directly impacting the accuracy of these navigation systems. This impact can be reduced by updating preoperative imaging using continuous information about the actual tumor position.

A safe and practical tracking technique to acquire real-time information on tumor position is electromagnetic (EM) tracking of EM sensors that are implanted in or around the tumor [9]. This technique does not require a radiation dose or line-of-sight, as opposed to alternatives such as intraoperative computational tomography (CT) and the use of optical markers [10–12]. EM tracking has been studied in a variety of fields, including surgery [13–17]. However, these EM tracking systems generally make use of wired sensors, which is not preferable for surgical applications. To avoid inconvenience for the patient prior to surgery, these wired sensors have to be placed intraoperatively, which hampers surgical workflow. Wires may also induce sensor migration and are prone to breaking. As an alternative to using wired sensors, a technique can be used to track wireless EM transponders. These transponders can be implanted into the tissue before surgery, using a biopsy needle, for example, and can then be visualized on preoperative imaging. This allows for preoperative registration of the EMTS with imaging, saving intraoperative time.

The only clinically cleared system available using this wireless EM tracking technique is the Calypso^®^ System (Varian Medical Systems Inc., Palo Alto, California, USA). This electromagnetic tracking system (EMTS), designed for radiation therapy (RT) applications, makes use of wireless EM transponders. For each transponder, 5-degrees-of-freedom (5DOF) information can be acquired, i.e., its position and orientation in 3D space, except for the rotation about the transponder axis (the sixth degree of freedom). These transponders are implanted through needles inside or close to the tumor [18]. When the transponders are excited by the EM field, they emit signals detected by a tracking sensor array (TA). This TA is able to track these transponders with an accuracy of < 0.5 mm root mean square error (RMSE) in an RT environment [19, 20] and with submillimeter accuracy in an OR environment [21].

Even though each transponder provides 5DOF information, the FDA-cleared EMTS uses only the combined translational (3DOF) information of the transponders to determine tumor orientations, and then 6DOF tumor information is available. By design, this system is also limited to tracking three transponders, i.e., the minimum amount of transponders required for achieving 6DOF with this method. Therefore, all transponders have to be implanted into the tissue and no transponders are available to track a surgical tool, required for surgical navigation. Updating the technical design of the EMTS may allow a tool to be tracked by additional transponders. However, since the sampling rate of the EMTS is limited to 25 Hz, adding transponders will strongly reduce the sampling rate per transponder. This will have a considerable effect on the refreshment rate of the navigation interface, thereby making it less intuitive and efficient. Alternatively, the transponder system can be combined with an optical tracking system (OTS) [22]. This is not preferable, because using a second tracking system reduces accuracy and an OTS requires line-of-sight.

In order to avoid using a second tracking system, without having to change the technical design of the EMTS, the 5DOF single transponder information can be used instead of only the 3DOF information. Then, one transponder can track a tooltip, leaving two 5DOF transponders to derive 6DOF tumor information. The accuracy of this 6DOF information is expected to depend on the transponder configuration. Ideally, the two transponders should be positioned under a 90° angle, but in clinical practice they are implanted through straight needles and preferably all through the same entry point. As a result, the angle between the two transponders will likely be relatively small, e.g., 0°–15°. The accuracy with which 6DOF data can then be obtained is highly dependent on the measurement accuracy of the EMTS [19–21], the angle and distance between the transponders, and how the coordinate system of the two combined transponders is defined. To our knowledge, the accuracy of the rotational component of single transponder 5DOF information has not been reported yet, even though 5DOF information has always been available for research purposes. The reason for this is that only translational accuracy of the 5DOF transponders is important for the FDA-cleared system, given its method to derive 6DOF.

The purpose of this study was to assess the feasibility of tumor tracking using two 5DOF wireless EM transponders, which is an important step in the development of wireless EM surgical navigation. Two methods, to derive 6DOF tumor motion information out of two 5DOF transponders, were defined and evaluated with an accuracy assessment. Results were compared to a Default method, using all three transponders, and an OTS was used as a reference for accuracy.

## Materials and methods

**Fig. 1:**
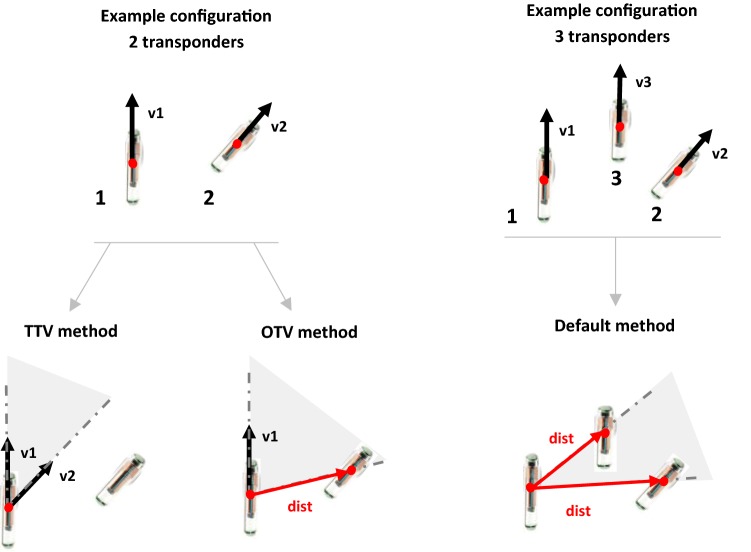
Illustration of how a plane (indicated with a light-gray area) is defined out of two 5-degrees-of-freedom transponders, using the TTV and OTV methods (left) and out of three transponders using the Default method (right). The black arrows v1, v2 and v3 represent the three transponder vectors of transponders 1, 2 and 3, respectively. The red arrows, dist, represent the 3D distance vectors between two transponder origins

The two methods to obtain 6DOF information out of two 5DOF transponders start with defining a plane, as illustrated in Fig. 1. Using the plane, the coordinate system, i.e., an EMTS object coordinate system, was determined. Using the 5DOF information, the transponders can be described as unit vectors with a position (i.e., the transponder origin) and an orientation in 3D space. When brought to the same origin, two of these transponder vectors create a plane. This is done in the first method and is referred to as the two transponder vectors (TTV) method. In the second method, the one transponder vector (OTV) method, the plane was defined using one of the transponder vectors in combination with the 3D distance vector from one transponder origin to the other. The OTV and TTV method were compared to the Default method in which all three transponders are used. This Default method defines a plane using the 3D distance vector between the origins of transponders 1 and 3 and the 3D distance vector between the origins of transponders 1 and 2 (Fig. 1).

For the OTV and TTV method, the *x*-axis of the EMTS object coordinate system was defined by one of the transponder vectors, v1 in Fig. 1. For the Default method, the *x*-axis is defined by the distance vector between transponders 1 and 3. Then, using the cross-product between the *x*-axis and transponder vector (TTV method) or 3D distance vector between transponders 1 and 2 (OTV and Default method), the *z*-axis was defined. The cross-product between the *z*-axis and *x*-axis then resulted in the *y*-axis.

The TTV method is expected to be less accurate when the angle is smaller between the two transponders, i.e., angle *α*. Any inaccuracies of the transponder vectors will then have more effect on the plane definition. Similarly, the OTV method is expected to be less accurate when the angle is smaller between the used transponder vector and the 3D distance vector, i.e., angle *β*. It is expected the accuracy of the OTV method is also dependent on the distance between the transponders, because the smaller this distance, the more inaccuracies of the transponders position information are expected to affect the 3D distance vector. Given all these dependencies, the worst-case scenario is when the two transponders are parallel, in-line, and have a minimal inter-transponder distance.

In this study, the OTV and TTV methods were evaluated for close to worst-case scenario transponder configurations. The assessed accuracy of both methods was compared to the accuracy of the Default method, and with the Northern Digital Polaris Spectra Optical Tracking System (OTS) as a reference for all methods. This OTS has a known mean positional and rotational accuracy of 0.185 ± 0.137 mm and 0.383° ± 0.183°, respectively [23].

### Measurement setup

**Fig. 2:**
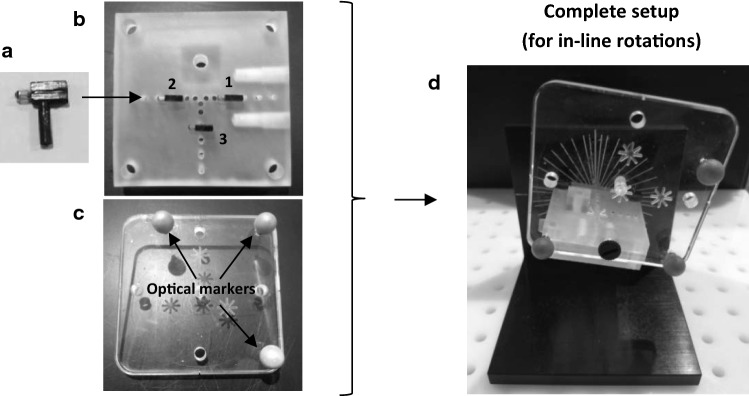
Pictures of the measurement setup. On the left, the transponder holder (**a**), the 3D-printed transponder plate with the three transponders (transponder numbers indicated) (**b**) and the OTS object that was mounted on the transponder plate (**c**). On the right, the assembly of the transponder plate and the OTS object within the complete setup (**d**) (setup for in-line rotations is shown)

**Fig. 3:**
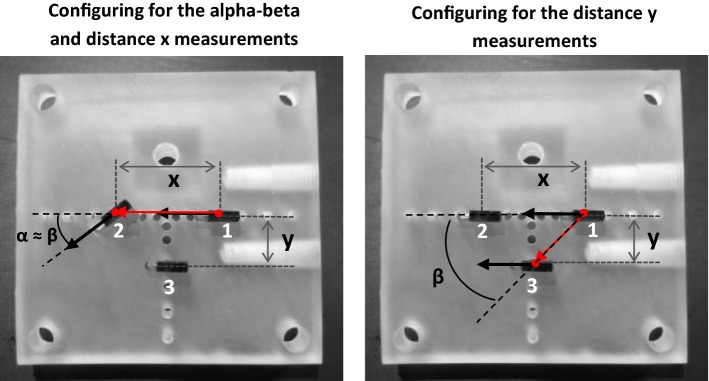
Illustrations of configuring transponders on the transponder plate. On the left, how the transponders were configured for the alpha–beta and distance *x* measurements. On the right, how the transponders were configured for the distance *y* measurements. The bold black arrows represent the transponder vectors, and the red arrow represents the 3D distance vector used for the OTV method. In the right illustration, only angle *β* is shown, angle *α* was 0.5° in these measurements

**Fig. 4:**
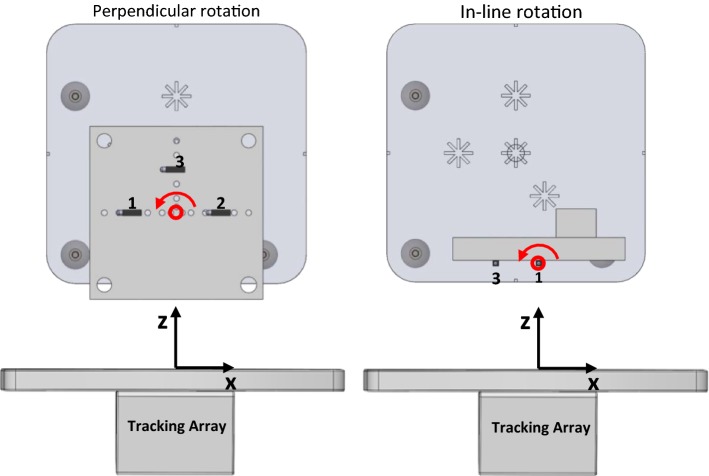
Illustrations of perpendicular (left) and in-line object rotation (right). Per rotation plane, the transponder plate is shown with the object of the optical tracking system attached to it, at object rotation angle *γ* = 0. The tracking array is shown on a smaller scale than the rotating object, but illustrates the global coordinate system of the electromagnetic tracking system with respect to the transponders (transponder numbers are indicated). The red circles indicate the point of rotation in both cases, and the red arrows indicate the direction of positive rotation

In the measurement setup, shown in Fig. 2, the object to be tracked was a small 3D-printed plate of 50 × 50 × 8 mm. The three transponders were fixed onto this transponder plate using transponder holders. By design, the transponders were in plane with the transponder plate surface and the transponder origins were in-line with the axes of the holes in which the transponder holders were inserted (accuracy between 0.05 and 0.1 mm). The transponder plate had two arrays of holes, all interspaced with 5 mm (accuracy of ± 0.2 mm). The long array allowed for adjusting distance *x* between transponders 1 and 2, as illustrated in Fig. 3. The short array allowed for adjusting the position of transponder 3 (distance *y*). Angle *α* could be manually adjusted by rotating the transponder holders about the axes of the holes. Angles *α* and *β* could be set equal for transponders 1 and 2 in this design, allowing for a direct comparison between the OTV and TTV methods. This was achieved by keeping transponder 1 in-line with the long array of holes and then only rotating transponder 2. After configuring the transponders, a transparent Perspex square plate (90 × 90 × 8 mm) with optical markers was mounted on the transponder plate so it could be tracked by the OTS (Fig. 2). Within the measurement setup, the transponder plate was restricted to two defined movements, i.e., either rotation about the axis of transponder 1 or rotation perpendicular to the transponders (Fig. 4). In each of these rotation planes, the object could be fixed at any object rotation angle, i.e., angle *γ*. The transponder plate had a fixed position with respect to the TA in the center of the EMTS field of view.

Three sets of transponder configurations were evaluated. In the first two sets, the accuracy was assessed for scenarios where transponders were in-line and angles *α* and *β* were small. In these sets, transponders 1 and 2 were used for the OTV and TTV methods, configuring them according to Fig. 3. In the first set, the distance between transponders 1 and 2 was fixed at *x* = 30 mm and the rotation of transponder 2 was varied such that angle *α* ≈ *β* = 1°, 2°, 5°, 10° and 45° (accuracy ± 0.5°). This set is referred to as the alpha–beta measurements. In the second set of measurements, angles *α* and *β* were both fixed at 5° and the distance between transponder 1 and 2 was set to *x* = 10, 20, 30 and 40 mm. This set is referred to as the distance *x* measurements. In these first two sets of measurements, transponder 3 (in these sets only used for the Default method) was positioned at half the inter-transponder distance, i.e., *y* = 0.5 * *x*, except for *x* = 10 mm where *y* was 10 mm.

In the third set, the accuracy was assessed for a scenario where the transponders were parallel but not in-line. In this set, transponders 1 and 3 were used for the OTV and TTV methods, configuring them according to Fig. 3. The distance between transponders 1 and 2 was fixed at *x* = 40 mm, while distance *y* was varied (*y* = 5, 10, 15 and 20 mm), with angle *α* between transponders 1 and 3 fixed at 0.5°. This measurement set is referred to as the distance *y* measurements.

All transponder configurations were evaluated for the two possible object rotations (perpendicular and in-line). Measurements started as illustrated in Fig. 4 (at *γ* = 0). This reference measurement was used to register the coordinate systems defined by the OTV, TTV and Default methods with the OTS object coordinate system. Subsequently, transponder plate rotation was adjusted and fixed to *γ* = − 5°, − 10°, − 20°, 0°, 5°, 10° and 20° (accuracy of ± 0.5°).

For each object orientation, 150 samples of data (OTS and EMTS) were recorded at a sampling rate of 9 Hz, using our custom-made software. All tracking information was communicated using OpenIGTLink TRANSFORM messages. PlusServer (https://plustoolkit.github.io/) was used to combine the OTS and EMTS data into one data stream [24]. The OpenIGTLink.dll from IGSTK (www.igstk.org) was used in the custom-made software to receive and translate the OpenIGTLink messages.

### Data analysis

For the data analysis, only orientation data were used, since positional information of the transponders has already been shown to be of submillimeter accuracy [19, 20], also in the OR environment [21]. Per sample of the reference measurement, the EMTS object coordinate systems were registered to the OTS object coordinate system. Then, the resulting registrations were averaged using the quaternion averaging method presented by Markley et al. [25]. This way, one registration was obtained for each of the methods (OTV, TTV and default), for that specific transponder configuration and rotation plane. These registrations describe relationships that are assumed to stay constant over all object rotation angles *γ*. Any deviations were defined as orientation errors. These errors are expressed in Tait–Bryan Euler angles of these matrices, according to a *ZYX* rotation sequence. The results for the OTV, TTV and Default methods are reported with box-and-whisker plots per measurement of 150 samples. The whiskers denote the 95% confidence interval (^95^CI), and outliers are excluded. Median errors < 1° and ^95^CI < 5° were considered acceptable.

## Results

**Fig. 5:**
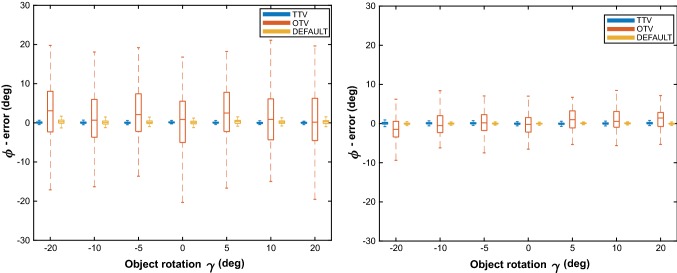
Boxplot results of the object orientation accuracies using the OTV, TTV and Default methods during in-line (left) and perpendicular rotations (right), where inter-transponder distance *x* = 10 mm and angle *α* = 5°. Error angle *ϕ* is plotted against object rotation angle *γ*

Figure 5 shows a typical example of the results of Euler error angle *ϕ*, i.e., the rotation error about the *x*-axis of the EMTS object coordinate system. The Default method was always accurate, with angle *ϕ* median errors < 0.2° and ^95^CI < 3.5°. For all methods, no clear effect of the object rotation angle *γ* on the object orientation accuracy has been observed. For the TTV method, the object orientation accuracy did not clearly differ between object rotation planes. The OTV method, on the other hand, did show differences between in-line and perpendicular rotation, where in general the jitter was higher for in-line rotations. However, independent of the object rotation plane, it was obvious that the TTV method performed better than or similar to the OTV method for all transponder configurations. The other Euler error angles, i.e., rotation errors about the *y*- and *z*-axes of the EMTS object coordinate system, were negligibly small in all measurements (median errors < 0.15°, ^95^CI < 0.6°).

**Fig. 6:**
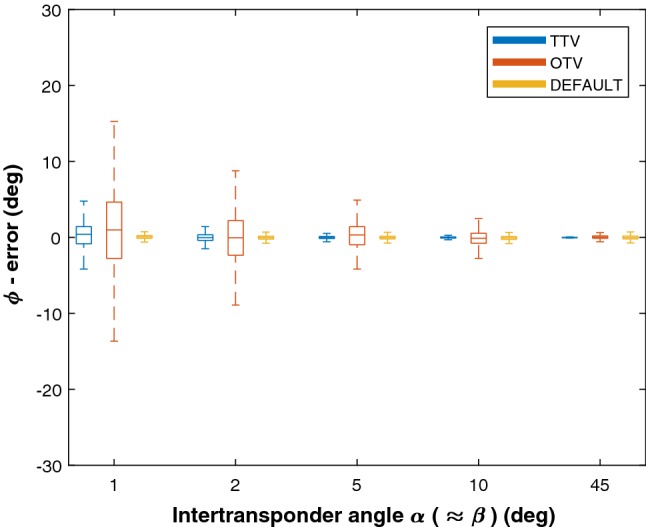
Boxplot results of the object orientation accuracies using the OTV, TTV and Default methods for different angles *α*, where distance *x* = 30 mm. Error angle *ϕ* is plotted against angle *α* (≈ *β*)

**Fig. 7:**
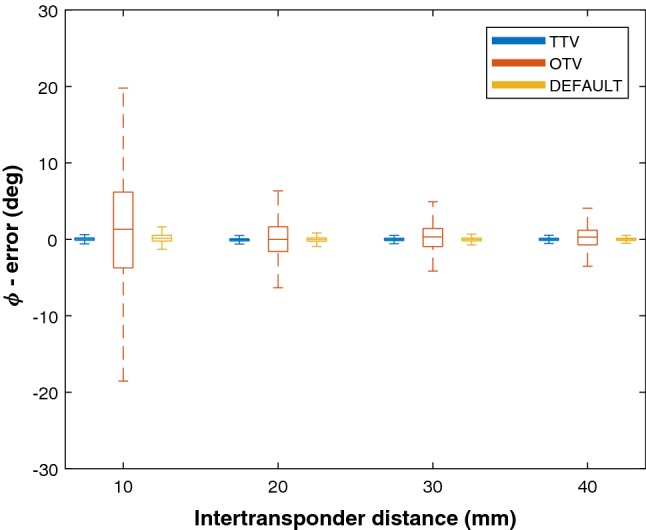
Boxplot results of the object orientation accuracies using the OTV, TTV and Default methods for different inter-transponder distance *x*, where angle *α* ≈ *β* = 5°. Error angle *ϕ* is plotted against distance *x*

**Fig. 8:**
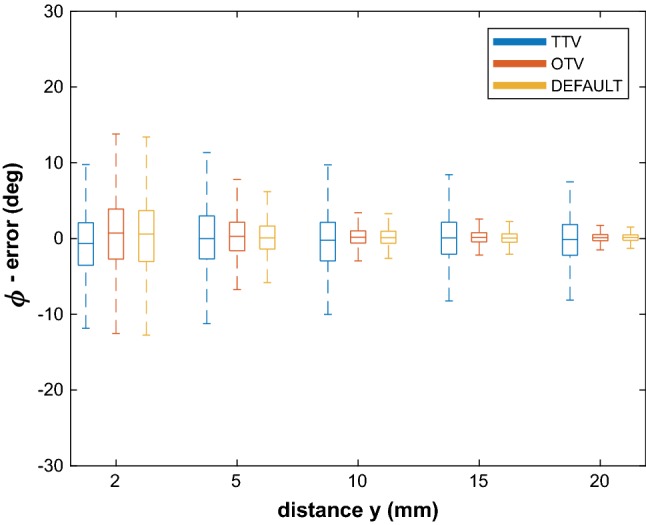
Boxplot results of the object orientation accuracies using the OTV, TTV and Default methods in the third set of measurements, where *x* = 40 mm, *α* = 0.5° and *y* was changed. Error angle *ϕ* is plotted against distance *y*

Based on the above findings, the rest of the analysis focused on Euler angle *ϕ* errors and only for in-line rotations, i.e., the object rotation plane with generally the largest errors. Also, per transponder configuration, the data of all object rotations *γ* together were analyzed. Therefore, the box plots in the results shown in Figs. 6, 7 and 8 each represent 750 samples.

The results of the alpha–beta measurements are plotted in Fig. 6. Both the TTV and OTV methods showed an increased object orientation accuracy when angles *α* and *β* increased. The TTV method performed better than the OTV method and was acceptable for angle *α* ≥ 2° (median errors < 0.5°, ^95^CI < 3°) and even more accurate than the Default method for angle *α* ≥ 5°. The OTV method had acceptable median errors of < 0.2°, but a ^95^CI of 6.5°, for *β* ≥ 10°. For *β* = 45°, the accuracy of the OTV method was acceptable (median error < 0.1° and ^95^CI < 2°) and as accurate as the Default method.

The results of the distance *x* measurements are plotted in Fig. 7. An increase in the accuracy of the OTV method was observed with increasing inter-transponder distance *x*. However, even for *x* = 40 mm, the ^95^CI still exceeded the acceptable level (^95^CI > 6°), whereas the median errors were acceptable for *x* ≥ 20 mm. The accuracy of the TTV method did not change with changing inter-transponder distance and was acceptable for all distances (median errors < 0.1°, ^95^CI < 2°).

Figure 8 shows the results of the distance *y* measurements, where *α* was 0.5°. The TTV method was inaccurate for all distances *y*. The OTV method on the other hand was more accurate with increasing distance *y* and acceptable for *y* = 20 mm when angle *β* = 45° (median error < 0.2°, ^95^CI < 5°). The results for the Default method were similar to the OTV method results.

## Discussion

In this study, two methods were presented to derive 6DOF tumor motion information out of two implanted 5DOF wireless EM transponders, aiming to achieve wireless EM surgical navigation (given that a third transponder tracks a tooltip). The TTV method, which uses the vector information of both transponders, performed best and was accurate for almost all transponder configurations (median errors < 0.5°, ^95^CI < 3°). Compared to the Default method, the TTV method performed similar or even better. Only close to parallelism (angle *α* < 2°), the TTV method was inaccurate. In these cases, the OTV method, which uses the vector information of one transponder and the distance vector between both transponder origins, may be the preferred option only when the transponders are side by side (angle *β* ≥ 45°) and the distance between their axes is at least 20 mm. In all other cases, when the transponders are more in-line and parallel (angle *α* < 2°), accurate orientation information cannot be obtained with two transponders.

In clinical practice, an angle of less than 2° between two implanted transponders is very unlikely. From that perspective, it can be assumed that the TTV method is always applicable for accurate tumor tracking. This gives great flexibility for the clinician implanting these transponders, because the transponder locations relative to each other do not have to be taken into account. Moreover, the inaccuracies observed were mostly due to jitter. Therefore, even if the transponders are approaching parallelism, the use of filtering may be an option for improving accuracy.

The rotational errors observed of the TTV method for angle *α* ≥ 2° were comparable to a study done by Wu et al. [26] who evaluated target tracking using all three transponders (mean error < 0.5° and standard deviations < 1.5°). This is in line with our findings for the Default method. The observed errors were also comparable to rotational errors found for wired alternatives [27, 28].

In the analysis, the orientation errors were defined by three Tait–Bryan Euler angles. However, only one Euler angle showed inaccuracies. This Euler angle described the rotation error about the *x*-axis of the coordinate system that was defined with the evaluated methods. In both methods, the orientation of the *x*-axis was directly derived from the transponder vector information of one transponder. This makes the *x*-axis independent of the configuration of both transponders, as opposed to the subsequently derived *y*- and *z*-axes. The biggest rotational errors are then to be expected in the plane that does not include the *x*-axis, i.e., the *y*–*z* plane, and therefore are about the *x*-axis. For the transponder orientations evaluated in this study, this finding also indicates that the transponder vector accuracy of a single transponder is high (median errors < 0.15° and ^95^CI < 0.6°). To our knowledge, this is a novel finding that has not yet been reported in the literature.

For both methods, rotating the object did not clearly affect the object orientation accuracy (Fig. 5). This indicates that the accuracy of the two methods is independent of tumor rotation and hence mainly dependent on the transponder configuration.

The reported results are obtained using an OTS as a reference, which has a rotational inaccuracy as well. Since the inaccuracy of the OTS is small, i.e., 0.383° ± 0.183° [23], the above conclusions still apply.

In the measurements, the transponders were always in the same plane, which is not likely to occur in clinical practice. However, this will not have an additional effect on the accuracy of the OTV and TTV methods, on top of inter-transponder distance (OTV) and transponder orientation (OTV and TTV).

The study was performed in a rigid setting, which did not take into account possible transponder migration or tumor deformation. Migration may cause the origin of the coordinate system, defined with the two transponders, to shift. Additionally, migration can also result in a change in angle between the transponders, resulting in a rotational error to the same degree. Studies have shown, however, that migration of these transponders is low in the pancreas (0.7 ± 1.1 mm) [29], and has not been encountered in post-prostatectomy patients [30] and prostate cancer patients [31], although some stabilization time may be required. Chances of migration can be further limited by using anchored transponders, designed for lung cancer treatment [32]. Whereas migration can be limited, tissue deformation is difficult to avoid. Tissue deformation may change the transponder configuration, especially during the surgical procedure. The effect of tissue deformation is dependent on the distance between the transponders and on their location and orientation with respect to the tumor. The further the transponders are apart, the more likely the transponder configuration changes due to tissue deformation. However, using the real-time relative translational and rotational information between the transponders, the deformation can be characterized to a certain extent. The navigation interface can then be updated accordingly, reducing the impact of the deformation on the accuracy. For short distances between the transponders, on the other hand, the transponder configuration is likely to be unaffected by tissue deformation. Distant tissue deformations may then be undetected, as well as local deformations of the tissue in which both transponders are implanted. In particular, these undetected local deformations are expected to have a big impact on the tumor border assessment accuracy distant from the transponders. Further research is required to find optimal transponder locations and orientations relative to the tumor tissue, to be able to detect tissue deformation and then reduce its effect on the navigation accuracy.

## Conclusion

Accurate target tracking with two 5DOF EM transponders is feasible, even for worst-case transponder configurations. The best method to derive 6DOF target information out of the two transponders is to use transponder vector information to define a plane and then a coordinate system. This method is robust for inter-transponder angles of 2° or more and independent of transponder locations with respect to each other. The provided results encourage further development of a surgical navigation approach where a readily available wireless transponder tracking system is used to track both the tumor and a surgical tool.

## References

[1] Zygomalas A, Kehagias I (2019). Up-to-date intraoperative computer assisted solutions for liver surgery. World J Gastrointest Surg.

[2] Galloway RL, Herrell SD, Miga MI (2012). Image-guided abdominal surgery and therapy delivery. J Healthc Eng.

[3] Takeuchi H, Kawakubo H, Takeda F, Omori T, Kitagawa Y (2012). Sentinel node navigation surgery in early-stage esophageal cancer. Ann Thorac Cardiovasc Surg.

[4] Kingham TP, Scherer MA, Neese BW, Clements LW, Stefansic JD, Jarnagin WR (2012). Image-guided liver surgery: intraoperative projection of computed tomography images utilizing tracked ultrasound. HPB.

[5] Senft C, Ulrich CT, Seifert V, Gasser T (2010). Intraoperative magnetic resonance imaging in the surgical treatment of cerebral metastases. J Surg Oncol.

[6] Ochs BG, Schreiner AJ, de Zwart PM, Stockle U, Gonser CE (2016). Computer-assisted navigation is beneficial both in primary and revision surgery with modular rotating-hinge knee arthroplasty. Knee Surg Sport Traumatol Arthrosc.

[7] Aschendorff A, Maier W, Jaekel K, Wesarg T, Arndt S, Laszig R, Voss P, Metzger M, Schulze D (2009). Radiologically assisted navigation in cochlear implantation for X-linked deafness malformation. Cochlear Implant Int.

[8] Bilhar RPO, de Lima DA, Leite JAD, Porto MA (2018). Accuracy of pedicle screw insertion: a comparison between fluoroscopic guidance and navigation techniques. Acta Ortopédica Bras.

[9] Franz AM, Haidegger T, Birkfellner W, Cleary K, Peters TM, Maier-Hein L (2014). Electromagnetic tracking in medicine—a review of technology, validation, and applications. IEEE Trans Med Imaging.

[10] Chang SS, Nakano T, Okamoto T (2016). Usefulness of intraoperative computer tomography-assisted thoracoscopic segmentectomy for small-sized lung cancer. Ann Thorac Cardiovasc Surg.

[11] Wild E, Teber D, Schmid D, Simpfendorfer T, Muller M, Baranski AC, Kenngott H, Kopka K, Maier-Hein L (2016). Robust augmented reality guidance with fluorescent markers in laparoscopic surgery. Int J Comput Assist Radiol Surg.

[12] Tringale KR, Pang J, Nguyen QT (2018). Image-guided surgery in cancer: a strategy to reduce incidence of positive surgical margins. Wiley Interdiscip Rev Syst Biol Med.

[13] Zhu M, Bharat S, Michalski JM, Gay HA, Hou WH, Parikh PJ (2013). Adaptive radiation therapy for postprostatectomy patients using real-time electromagnetic target motion tracking during external beam radiation therapy. Int J Radiat Oncol Biol Phys.

[14] Braide K, Lindencrona U, Welinder K, Götstedt J, Ståhl I, Pettersson N, Kindblom J (2018). Clinical feasibility and positional stability of an implanted wired transmitter in a novel electromagnetic positioning system for prostate cancer radiotherapy. Radiother Oncol.

[15] Zhang H, Banovac F, Lin R, Glossop N, Wood BJ, Lindisch D, Levy E, Cleary K (2006). Electromagnetic tracking for abdominal interventions in computer aided surgery. Comput Aided Surg.

[16] Wagner M, Gondan M, Zollner C, Wunscher JJ, Nickel F, Albala L, Groch A, Suwelack S, Speidel S, Maier-Hein L, Muller-Stich BP, Kenngott HG (2016). Electromagnetic organ tracking allows for real-time compensation of tissue shift in image-guided laparoscopic rectal surgery: results of a phantom study. Surg Endosc.

[17] Ungi T, Gauvin G, Lasso A, Yeo CT, Pezeshki P, Vaughan T, Carter K, Rudan J, Engel CJ, Fichtinger G (2016). Navigated breast tumor excision using electromagnetically tracked ultrasound and surgical instruments. IEEE Trans Biomed Eng.

[18] Murphy MJ, Eidens R, Vertatschitsch E, Wright JN (2008). The effect of transponder motion on the accuracy of the calypso electromagnetic localization system. Int J Radiat Oncol Biol Phys.

[19] Balter JM, Wright JN, Newell LJ, Friemel B, Dimmer S, Cheng Y, Wong J, Vertatschitsch E, Mate TP (2005). Accuracy of a wireless localization system for radiotherapy. Int J Radiat Oncol Biol Phys.

[20] Franz AM, Schmitt D, Seitel A, Chatrasingh M, Echner G, Oelfke U, Nill S, Birkfellner W, Maier-Hein L (2014). Standardized accuracy assessment of the calypso wireless transponder tracking system. Phys Med Biol.

[21] Eppenga R, Kuhlmann K, Ruers T, Nijkamp J (2018). Accuracy assessment of wireless transponder tracking in the operating room environment. Int J Comput Assist Radiol Surg.

[22] Janssen N, Eppenga R, Peeters MJV, van Duijnhoven F, Oldenburg H, van der Hage J, Rutgers E, Sonke JJ, Kuhlmann K, Ruers T, Nijkamp J (2018). Real-time wireless tumor tracking during breast conserving surgery. Int J Comput Assist Radiol Surg.

[23] Wiles AD, Thompson DG, Frantz DD (2004). Accuracy assessment and interpretation for optical tracking systems. Proc SPIE.

[24] Lasso A, Heffter T, Rankin A, Pinter C, Ungi T, Fichtinger G (2014). PLUS: open-source toolkit for ultrasound-guided intervention systems. IEEE Trans Biomed Eng.

[25] Markley F, Cheng Y, Crassidis JL, Oshman Y (2007). Averaging quaternions. J Guid Control Dyn.

[26] Wu J, Ruan D, Cho B, Sawant A, Petersen J, Newell LJ, Cattell H, Keall PJ (2012). Electromagnetic detection and real-time DMLC adaptation to target rotation during radiotherapy. Int J Radiat Oncol Biol Phys.

[27] Nijkamp J, Schermers B, Schmitz S, de Jonge S, Kuhlmann K, van der Heijden F, Sonke JJ, Ruers T (2016). Comparing position and orientation accuracy of different electromagnetic sensors for tracking during interventions. Int J Comput Assist Radiol Surg.

[28] Maier-Hein L, Franz AM, Birkfellner W, Hummel J, Gergel I, Wegner I, Meinzer HP (2012). Standardized assessment of new electromagnetic field generators in an interventional radiology setting. Med Phys.

[29] Shinohara ET, Kassaee A, Mitra N, Vapiwala N, Plastaras JP, Drebin J, Wan F, Metz JM (2012). Feasibility of electromagnetic transponder use to monitor inter- and intrafractional motion in locally advanced pancreatic cancer patients. Int J Radiat Oncol Biol Phys.

[30] Canter D, Kutikov A, Horwitz EM, Greenberg RE (2011). Transrectal implantation of electromagnetic transponders following radical prostatectomy for delivery of IMRT. Can J Urol.

[31] Kupelian P, Willoughby T, Litzenberg D, Sandler H, Roach M, Levine L, van Waardenburg M, Cunningham A, Meeks S (2005). Clinical experience with the Calypso^®^ 4D localization system in prostate cancer patients: implantation, tolerance, migration, localization and real time tracking. Int J Radiat Oncol.

[32] Shah AP, Kupelian PA, Willoughby TR, Meeks SL (2011). Expanding the use of real-time electromagnetic tracking in radiation oncology. J Appl Clin Med Phys.

